# Anomalous Separation of Small Y-Chromosomal DNA Fragments on Microchip Electrophoresis

**DOI:** 10.3390/scipharm84030507

**Published:** 2016-05-26

**Authors:** Mohammad Jabasini, Ashraf Ewis, Youichi Sato, Yutaka Nakahori, Yoshinobu Baba

**Affiliations:** 1 Department of Pharmaceutical Information Science, Institute of Health Biosciences, The University of Tokushima, 1-78-1 Sho-machi, 770-8505 Tokushima, Japan; sato@ph.tokushima-u.ac.jp; 2 Department of Human Genetics Public Health, School of Medicine, The University of Tokushima, 3-18-15 Kuramoto-cho, 770-8503 Tokushima, Japan; ashraf_ewis@yahoo.com (A.E.); nakahori@basic.med.tokushima-u.ac.jp (Y.N.); 3 Department of Applied Chemistry, Graduate School of Engineering, Nagoya University, Furo-cho, Chikusa-ku, 464-8603 Nagoya, Japan; babaymtt@apchem.nagoya-u.ac.jp; 4 ImPACT Research Center for Advanced Nanobiodevices., Nagoya University, Furo-cho, Chikusa-ku, 464-8603 Nagoya, Japan; 5 Health Research Institute, National Institute of Advanced Industrial Science and Technology (AIST), Hayashi-cho 2217-14, 761-0395 Takamatsu, Japan

**Keywords:** anomalous separation, DNA, microchip electrophoresis

## Abstract

We investigated an anomalous DNA separation where two DNA fragments from the human Y-chromosome sY638 (64 bp) and sY592 (65 bp), with only one base pair difference, were separated. This result is abnormal since in a previous study, we found that 5 bp was the minimum difference between two DNA fragments that the microchip electrophoresis system can separate. The formation of a mini-loop in the structure of the DNA fragment of sY638 (64 bp) was strongly expected to be the reason. To investigate this, we synthesized three modified DNA fragments for sY638 (64 bp), and the modifications were in two expected locations for possible mini-loop formation. Later, the separation between sY592 (65 bp) and the three modified fragments of sY638 (64 bp) was not possible. Thus, we conclude that the formation of a mini-loop in the structure of the DNA is the reason behind this anomalous separation.

## 1. Introduction

The regular duplex structure with canonical A–T and G–C is the common structure for DNA. In addition to this, DNA can fold in several varieties of structures, and these structures have been observed, studied, and divided into several categories such as: hairpins, triplexes, and quadruplexes. These repeats can occur in the eukaryotic genome [1,2,3,4,5,6] and are called minisatellites. Among them, DNA hairpins have received remarkable attention because of their possible implications in several biological processes. In the last decade, many DNA hairpin structures have been determined [7] and they are involved in the biological processes in both prokaryotic and eukaryotic cells [8]; these hairpins have also been documented in replication origins [9,10].

Since the early 1990s, only DNA hairpins with mini-loops of two residues have been studied [11,12,13] and classified into different types according to their conformations [14,15], with the possibility that the two-base loop can occur in vivo too [16]. Also, it has been suggested that these mini-loops may play roles in gene regulation, recombination, or mutagenesis [17].

On the analytical side, capillary electrophoresis (CE) has played an important role for DNA analysis and genome sequencing. The minimized version of capillary electrophoresis and microchip electrophoresis offers many advantages for DNA analysis including ease of operation, fast separation, and low consumption of samples and reagents. Microchip electrophoresis is an important tool in genomic and proteomic analysis which will result in a new class of drugs based on gene therapy and DNA diagnosis.

In this study, we investigated an abnormal separation for DNA markers on microchip electrophoresis, and we searched for the reason standing behind this anomalous separation by studying the structure of the DNA markers, expecting that the formation of a mini-loop is the reason for it.

## 2. Materials and Methods

### 2.1. Microchip System

Separation was performed on the Agilent 2100 Bioanalyser (Agilent Technologies, Waldbronn, Germany) which has epifluorescent detection with a semiconductor laser which emits at 630 nm. Each chip has 12 sample wells with three gel-dye mix wells and one well for the ladder, with a depth of 10 μm, a width of 50 μm, and effective separation length of 15 mm, and it is made from soda lime glass, more information can be found at [18].

### 2.2. Samples

The DNA samples were obtained from Prof. Nakahori‘s lab, School of Medicine at the University of Tokushima, and it was prepared according to the method described in [19].

The three modified samples of sY638 (64 bp) were synthesized by Invitrogen, Corp. (Carlsbad, CA, USA), the modifications are as following:

(1) Modified sY638: CAGCAG has been modified to be GTCCAG, and it is called: 1-Modified sY638
(2) Modified sY638: TGTG has been modified to be ATTG, and it is called: 2-Modified sY638
(3) Modified sY638: The third modification contains both previous modifications, and it is called: 3-Modified sY638 (Table 1).

## 3. Results and Discussion

Exploring the characteristics of the separation of microchip electrophoresis is very important for DNA analysis on a chip. Thus DNA separation and analysis on microchip electrophoresis was subjected to several studies. Early studies focused on the validation of the microchip separation ability with special concerns including the separation time, the resolution of the separation on chip, the required volume of the sample, and the ability to separate standard markers as well as real samples [20,21,22]. The positive results obtained from these studies paved the way for a wide use of microchip electrophoresis in several DNA research studies. Moreover, the CE technology found several applications in DNA analysis by either using the chip technology alone or in combination with other technologies such as polymerase chain reaction (PCR), where the PCR is performed for the DNA markers then followed by microchip separation [23].

**Figure 1:**
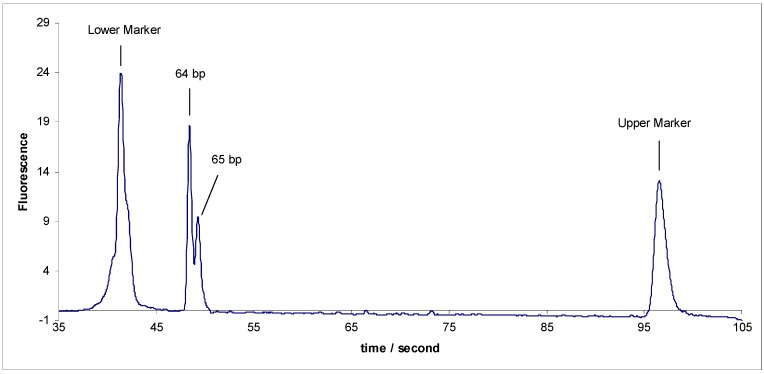
The anomalous separation of sY592 (65 bp) and sY638 (64 bp) before modification.

In a previous work, we studied the range of separation for DNA on a chip and we could separate two DNA markers with a length difference of only 5 bp, in a DNA range less than 100 bp. All other attempts to separate any two DNA fragments with a difference lower than 5 base pairs proved to be impossible, so we concluded that the 5 bp difference in the fragment length is the minimum size difference that the microchip could separate [21]. Recently, we separated two DNA markers with 1 bp difference sY638 (64 bp) and sY592 (65 bp). This result was repeatable (*n* = 12) and reproducible (Figure 1). Thus, this unexpected result encouraged us to explore more about this result since there is no existing data in the literature or reports from other research groups showing such a critical separation on a chip with one base pair difference.

Therefore, we tried to explore the possibility of separating two DNA fragments with a 3 base pair difference in the same DNA size range (less than 100 bp). The two DNA fragments were sY638, which consists of 64 bp, and sY610, which consists of 61 bp. Anyway, all the trials to separates these two fragments with the 3 bp length difference proved to be impossible.

These contradicting results led us to investigate the reason and possible explanation for this abnormal separation of fragments with one base pair difference.

**Figure 2:**
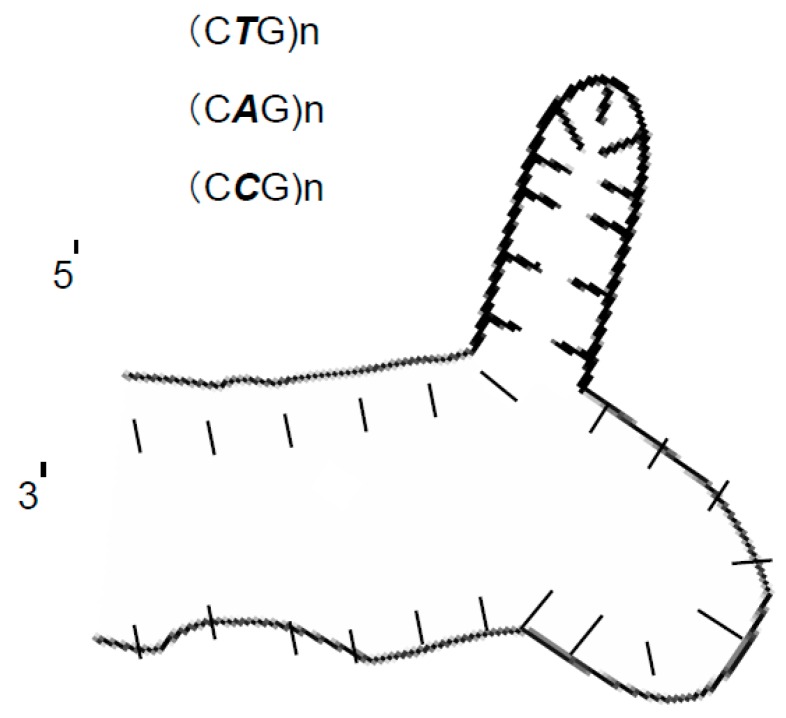
Hairpin DNA structure which can be formed from (CTG)n, (CAG)n, (CCG)n, (CGG)n repeats.

The possibility of the formation of a hairpin with a mini-loop in these DNA markers has been proposed as the possible explanation for the current abnormal separation. The hairpin with a mini-loop structure is a secondary structure in a nucleic acid molecule in which complementary sequences within the same strand anneal, forming a double strand stem while nucleotides between the paired regions form an unpaired, single-stranded loop. This unpaired single strand loop (the mini-loop) structure can be formed from triplet nucleotide repeats such as (CCG)n, (CTG)n, (CAG)n. This secondary structure is generally associated with triplet repeats and it has different formations; (A) Hairpin structure, which can be formed from (CTG)n, (CAG)n, (CCG)n, and (CGG)n repeats. Two CG bp are followed by either a T–T, A–A, C–C, or G–G mispair; (B) Slipped strand structure, which can be formed after denaturation of the repeated DNA sequences, if the repeated tract is renatured out of register. This can lead to a hairpin in opposite strands; (C) Folded slipped structure, which can be formed within a triplet repeat where it can be organized into a folded-type structure in which the single-stranded DNA within the loop of the hairpin can engage in the Watson-Crick-type hydrogen bonding [24,25,26,27,28]. In addition to these, some papers also reported the possibility of a hairpin with a mini-loop formation for only two base repeats [17]. The triplet repeats are associated with several genetic diseases such as myotonic dystrophy, fragile X syndrome, Huntington disease, several spinocerebellar ataxias, and Friedreich ataxia. In our samples, the secondary structure formation was expected to occur in the sY638 (64 bp) DNA fragment, since this would make sY638 (64 bp) a little smaller than usual and thus, it will be able to be separated from sY592 (65 bp). The possibilities of the formation of a secondary structure of a hairpin with a mini-loop in these DNA markers between the paired regions form an unpaired, single-stranded loop (Figure 2). Accordingly, the best method to investigate this possibility is to modify the DNA sequence of the fragment where the mini-loop is expected to be formed. To investigate this, the sequence of the sY638 (64 bp) DNA marker was studied, searching for any possible repeats where the formation of a loop is expected. Two repeats were found in the sequence of sY638 (64 bp): TG TG and CAG CAG (Table 1). In order to break the expected mini-loop formation, we synthesized three DNA fragments with similar nucleotide sequencing of sY638 (64 bp) where it was modified at the proposed nucleotides for the suspected mini-loops.

Three modified DNA fragments of sY638 (64 bp) were synthesized as mentioned in the “Experimental” section.

**Figure 3:**
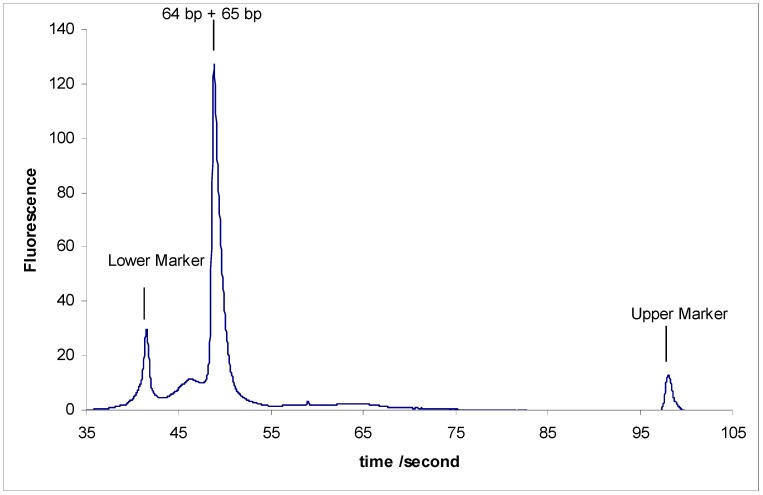
Separation results of sY592 (65 bp) and 1-Modified sY638 (64 bp).

After synthesizing the above modified DNA fragments, three separation experiments were carried out:

(1) Separation of sY592 (65 bp) from 1-Modified sY638 (64 bp), the separation did not occur.
(2) Separation of sY592 (65 bp) from 2-Modified sY638 (64 bp), the separation did not occur.
(3) Separation of sY592 (65 bp) from 3-Modified sY638 (64 bp), the separation did not occur either (Figure 3).

None of the fragments in the above three experiments were able to be separated.

The inability to separate sY592 (65 bp) from any of 1-Modified sY638 (64 bp), 2-Modified sY638 (64 bp), and 3-Modified sY638 (64 bp) strongly supports the supposition that the reason behind this 1 bp abnormal separation is a mini-loop formation in one of these two base pair repeats or in both of them. The formation of this mini-loop in sY638 (64 bp) makes its length lowerthan usual standard length and thus enables it to be separated from sY592 (65 bp).

This mini-loop was broken when the sequence was modified and thus, this unexpected phenomenon disappeared. Accordingly, we can say that the mini-loop formation is behind this anomalous DNA separation, and when the fragment length was preserved by the performed nucleotide sequence modifications, the mini-loop formation was eliminated and the anomalous separation disappeared.

**Figure 4:**
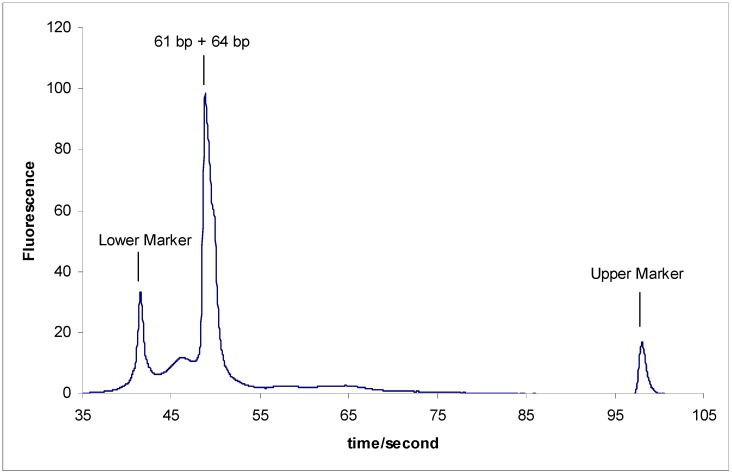
Separation results of the sY610 (61 bp) and 1-modified sY638 (64 bp).

Later, and for a double check, we tried to separate sY610 (61 bp) from the modified fragments of sY638 (64 bp) to check the possibility of separating two DNA fragments with a difference lowerthan 5 bp. All the modified fragments of sY638 (64 bp) were not able to be separated from sY610 (61 bp) (Figure 4).

Accordingly, we can conclude that:

- The inability to separate sY610 (61 bp) from any of the modified fragments of sY638 (64 bp) supports a previous study result that 5 bp is the minimum length difference for separating two DNA marker fragments on the microchip electrophoresis system [21].
- This anomalous separation can be a guiding example for the researcher in the separation field that if any research study resulted in separating two DNA markers with a very limited difference between the two DNA markers This result must not be accepted directly and should be deeply studied by taking into consideration that an anomalous DNA structure might be behind that.

## Figures and Tables

**Table 1:** sY638 (64 bp), the first and the second rows are the fragments without modification (the expected mini-loop places are in bold). Other rows are the three modified sY638 (64 bp) with its complementary sequence (the modification sites are underlined).

The Fragment	The Sequecing
SY638 (64 bp) without modification	5′-GACCACAAGA AAAC**TGTG**AG TGGCTTTCAG AAACTTGAGA AACTGGACCC TATTG**CAGCA G**ATC-3′
SY638 (64 bp) without modification, complementary	5′-GAT**CTGCTG**CAATA GGGTCCAGTT TCTCAAGTTT CTGAAAGCCA CT**CACA**GTTTTCTTGTGGTC-3′
1-Modified SY638 (64 bp)	5′-GACCACAAGA AAACTGTGAG TGGCTTTCAG AAACTTGAGA AACTGGACCC TATTGGTCCA GATC-3′
1-Modified SY638 (64 bp) complementary	5′-GATCTGGACC AATAGGGTCC AGTTTCTCAA GTTTCTGAAA GCCACTCACA GTTTTCTTGT GGTC-3′
2-Modified SY638 (64 bp)	5′-GACCACAAGA AAACATTGAG TGGCTTTCAG AAACTTGAGA AACTGGACCC TATTGCAGCA GATC-3′
2-Modified SY638 (64 bp) complementary	5′-GATCTGCTGC AATAGGGTCC AGTTTCTCAA GTTTCTGAAA GCCACTCAAT GTTTTCTTGT GGTC-3′
3-Modified SY638 (64 bp)	5′-GACCACAAGA AAACATTGAG TGGCTTTCAG AAACTTGAGA AACTGGACCC TATTGGTCCA GATC-3′
3-Modified SY638 (64 bp) complementary	5′-GATCTGGACC AATAGGGTCC AGTTTCTCAA GTTTCTGAAA GCCACTCAAT GTTTTCTTGT GGTC-3′
